# The Illinois Homœopathists

**Published:** 1867-06

**Authors:** 


### *The Illinois Homœopathists*

Are at loggerheads as much as their Parisian *confreres*, noticed on another page. A "Doctor" Miller tried hard to get Hahnemannic homœopathy, "pure and simple," adopted, in a recent conclave of the infinitesimals in this city—but the *Lord*, who was Chairman of the Committee on Materia Medica, did not smile on him in any wise. *Ecce signum*, (*Tribune* report):

"Dr. Lord, appointed at the last meeting a committee to report on Materia Medica, read a paper, stating that, though in advance of its rivals, it was a ponderous, unwieldy mass of incongruous matter. Its study was difficult and repulsive. Furthermore, in their opinion, the inappreciables and the infinitesimals are not far from the mark. The smaller the quantity of medicine given the better. In their opinion, all stomach symptoms of drugs were worthless, and all other symptoms valueless. The committee proceeded to destroy all faith in mince pie, by enumerating the forty-five symptoms resulting from the introduction of a piece into the stomach, which symptoms embrace that peculiar sensation as if the hypogastrium was twisting around the bladder."

Whatever resolutions are passed to the contrary, it is notorious that in this city the majority, if not all, of the fraternity use not only "appreciable" but "massive doses habitually. The *homœopathos* dodge is shrewd but scarcely laudable.

